# Assessing within‐subject rates of change of placental MRI diffusion metrics in normal pregnancy

**DOI:** 10.1002/mrm.29665

**Published:** 2023-05-15

**Authors:** Daniel Cromb, Paddy J. Slator, Miguel De La Fuente, Anthony N. Price, Mary Rutherford, Alexia Egloff, Serena J. Counsell, Jana Hutter

**Affiliations:** ^1^ Centre for the Developing Brain, School of Biomedical Engineering and Imaging Sciences King's College London London UK; ^2^ Centre for Medical Image Computing, Department of Computer Science University College London London UK; ^3^ Centre for Medical Engineering School of Biomedical Engineering and Imaging Sciences, King's College London London UK; ^4^ MRC Centre for Neurodevelopmental Disorders King's College London London UK

**Keywords:** diffusion imaging, longitudinal imaging, placental MRI, $T_{2}^{*}$ relaxometry

## Abstract

**Purpose:**

Studying placental development informs when development is abnormal. Most placental MRI studies are cross‐sectional and do not study the extent of individual variability throughout pregnancy. We aimed to explore how diffusion MRI measures of placental function and microstructure vary in individual healthy pregnancies throughout gestation.

**Methods:**

Seventy‐nine pregnant, low‐risk participants (17 scanned twice and 62 scanned once) were included. T_2_‐weighted anatomical imaging and a combined multi‐echo spin‐echo diffusion‐weighted sequence were acquired at 3 T. Combined diffusion–relaxometry models were performed using both a $T_{2}^{*}$‐ADC and a bicompartmental $T_{2}^{*}$‐intravoxel‐incoherent‐motion ($T_{2}^{*}\;\text{IVIM}$) model fit.

**Results:**

There was a significant decline in placental $T_{2}^{*}$ and ADC (both *P* < 0.01) over gestation. These declines are consistent in individuals for $T_{2}^{*}$ (covariance = −0.47), but not ADC (covariance = −1.04). The $T_{2}^{*}\;\text{IVIM}$ model identified a consistent decline in individuals over gestation in $T_{2}^{*}$ from both the perfusing and diffusing placental compartments, but not in ADC values from either. The placental perfusing compartment fraction increased over gestation (*P* = 0.0017), but this increase was not consistent in individuals (covariance = 2.57).

**Conclusion:**

Whole placental $T_{2}^{*}$ and ADC values decrease over gestation, although only $T_{2}^{*}$ values showed consistent trends within subjects. There was minimal individual variation in rates of change of $T_{2}^{*}$ values from perfusing and diffusing placental compartments, whereas trends in ADC values from these compartments were less consistent. These findings probably relate to the increased complexity of the bicompartmental $T_{2}^{*}\;\text{IVIM}$ model, and differences in how different placental regions evolve at a microstructural level. These placental MRI metrics from low‐risk pregnancies provide a useful benchmark for clinical cohorts.

## 1 INTRODUCTION

The placenta delivers oxygen and nutrients to the developing fetus and removes waste products of fetal metabolism. Comprehensive assessment of normal placental development throughout pregnancy is important to better understand and identify atypical development, such as that seen in preeclampsia (PE),^1^, ^2^ pregnancies affected by intrauterine growth restriction (IUGR),^3^ or in the presence of fetal abnormalities such as congenital heart disease.^4^

Placental MRI is a safe, noninvasive technique, suitable for larger maternal body habitus and later gestational ages (GAs), which can be used to generate useful metrics of placental function and microstructure during pregnancy.^5^, ^6^, ^7^ It produces objectively interpretable imaging data that can account for the dynamic nature of this organ.^8^, ^9^

Most research in quantitative placental MRI to date has relied on either $T_{2}^{*}$ mapping as a proxy for placental function^10^, ^11^ or diffusion imaging techniques to probe the microstructure of the placenta.^12^, ^13^, ^14^

$T_{2}^{*}$ relaxometry exploits the BOLD effect linking a shorter $T_{2}^{*}$ value to, among other factors such as geometry and the distribution of blood within the tissue being studied, a higher concentration of deoxygenated hemoglobin. Data are acquired using gradient echo MR sequences at different TEs and the decay in $T_{2}^{*}$ signal is analyzed using data‐fitting techniques. The relationship between decreased placental $T_{2}^{*}$ with advancing gestation and in pregnancy complications such as preeclampsia,^2^, ^15^ fetal growth restriction,^16^ and low birth weight^17^ are well established.

Placental diffusion MRI (dMRI) offers an opportunity to investigate the microstructure of the placenta by studying the ADC of the tissues being imaged. Based on its sensitivity to the Brownian motion of water, the ADC provides in vivo information about the density of tissue as well as its micro‐architecture, such as anisotropic structures. For this, data are acquired at a range of *b*‐values and b‐vectors, with a variety of models available to derive quantitative metrics related to the microstructural properties of underlying biological tissues. One of these models, the intravoxel incoherent motion (IVIM) model,^18^ allows extraction of diffusion‐weighted signal from different regions within the placenta, corresponding to perfusing (as a proxy for faster flowing “pseudo‐diffusing” blood) or diffusing (as a proxy for slower flowing “truly diffusing” blood) compartments. This serves as a useful tool to probe the underlying tissue and vascular properties of the placenta, observing how they alter across gestation, or in pathology.

Current studies using dMRI to investigate normal placental development are, however, limited by two challenges:

Firstly, most involve assessing either function or microstructure separately but rarely describe both of these characteristics in the same placenta. However, the complex interactions between structural and functional properties at the core of placental function is influenced by both the microstructure of the villous trees and the properties of the intervillous space, and therefore calls for more comprehensive assessments. Individual contrasts fall short of reflecting this, and combined dMRI scans have therefore recently gained interest, assessing oxygenation and microstructure simultaneously and providing a larger sampled parameter space for more advanced analysis techniques.^1^, ^19^, ^20^

Secondly, the majority of previous studies are cross‐sectional, reporting how MRI measures of placental function or microstructure change throughout pregnancy in different subjects imaged at different times. Resulting “normal” curves illustrating the behavior of placental properties over gestation therefore fail to inform about the extent of normal *within‐subject* variability, making it difficult to investigate the robustness of such measures. Understanding the trajectory of various measures of placental development for individual pregnancies may aid prediction of when clinical intervention is necessary, helping to guide the use of subsequent investigations.

### 1.1 Aims

The primary aim of this study was to use data acquired from participants undergoing two scans in the same pregnancy to generate within‐subject rates of change of these dMRI placental metrics in order to assess the variability of these measures in individual pregnancies. Secondary aims were to: (i) use an efficient multi‐modal pulse sequence, together with a comprehensive voxel‐wide analysis technique of the placenta, to explore how diffusion MRI (dMRI)–derived metrics of placental function ($T_{2}^{*}$) and microstructure (ADC) within‐subject change in normal pregnancy; and (ii) demonstrate the reliability of such efficient multi‐modal measures of placental function.

## 2 METHODS

### 2.1 Ethics statement

**FIGURE 1:**
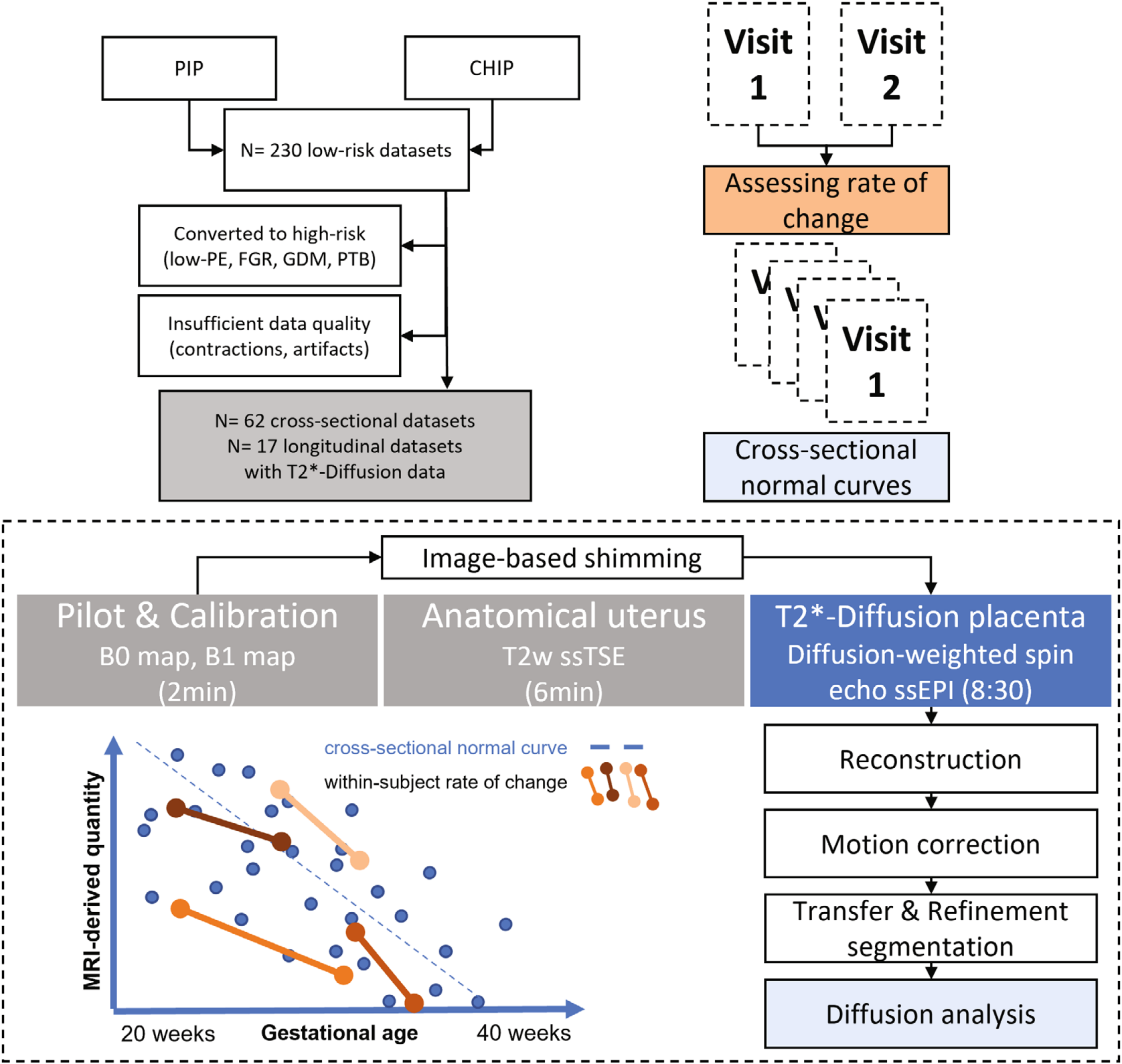
Study overview displaying the cross‐sectional and within‐subject evaluation, the flow chart of participants, and the acquisition and analysis pipelines

The data for this study was acquired as part of ethically approved studies (Congenital Heart Disease Imaging Project [REC 21/WA/0075] and Placental Imaging Project [REC16/LO/1573]) between 2017 and 2022. The data flow and study overviews are shown in Figure 1. Participants recruited to the Placental Imaging Project underwent a single MRI scan during pregnancy and constitute the cross‐sectional cohort. Participants recruited to the Congenital Heart Disease Imaging Project underwent two scans during pregnancy and constitute the longitudinal cohort. The same MRI protocol was applied for both cohorts, with details specified below.

### 2.2 Inclusion criteria

Placental data from these studies were included in this analysis if the GA at the time of the scan was over 20 weeks, and if the pregnancy was considered low risk, with the absence of PE, fetal growth restriction, or gestational diabetes at the time of recruitment and scanning. Scans were subsequently excluded if the pregnancy resulted in a delivery before 37 weeks GA, if PE, fetal growth restriction, or gestational diabetes were newly diagnosed between scan and delivery, or if any significant incidental fetal or placental findings were reported on imaging. Data sets with insufficient quality, that is, cropping of the placenta, or visible contractions at any time during the scan were also excluded.

This resulted in a total of 17 paired longitudinal datasets, defined as two scans in the same pregnancy (34 scans in total), and 62 cross‐sectional datasets.

### 2.3 Participant preparation

After informed, written consent was obtained, imaging was performed on a clinical Philips Achieva 3 T magnet scanner using a cardiac 32‐channel cardiac surface coil. All imaging was performed in supine position with frequent verbal interaction between radiographers and participants, continuous assessment of maternal blood oxygen saturation levels and heart rate, plus blood pressure measurements at 10 min intervals. The total scan time was limited to 30 min with a break in the middle. All sequences were individually assessed and complied with the requirements of safe fetal MRI, as previously published.^21^ Noise‐canceling headphones were provided for maternal comfort.

### 2.4 Image acquisition

Following the pilot scan and B_0_ and B_1_ calibration scans, anatomical imaging using T_2_‐weighted turbo‐spin‐echo sequences and a combined $T_{2}^{*}$‐diffusion scan were performed as depicted in Figure 1. For this combined $T_{2}^{*}$‐diffusion scan using a technique referred to as *ZEBRA*,^1^, ^21^ the diffusion‐weighted spin‐echo scan was extended to include four TEs after each diffusion‐weighting was performed, here at 78, 114, 150, and 186 ms. The repeatability of the $T_{2}^{*}$‐diffusion sequence has been demonstrated as a first step previously in MR phantom and adult brain studies.^21^ To further investigate in vivo repeatability of the $T_{2}^{*}$‐diffusion sequence and image processing pipeline also in vivo, this sequence was repeated at the end of a scanning session for four participants in the longitudinal cohort. The details of this experiment and the results are described in the Supporting material (Table S1, Figure S1). The chosen diffusion preparations were maintained from a previous study optimizing them for the properties of the human placenta.^22^ This resulted in three rotating diffusion gradient directions being used at *b* = [5, 10, 25, 50, 100, 200, 400, 600, 1200, 1600] s mm^−2^, eight directions at *b* = 18 s mm^−2^, seven at *b* = 36 s mm^−2^, and 15 at *b* = 800 s mm^−2^ (Table 1). The choice of the gradients was performed to avoid directional bias as described in Slator et al.^22^

**TABLE 1:** Scan parameters for the considered functional sequences: multi‐echo gradient echo EPI and multi‐echo diffusion‐weighted EPI

**Multi‐echo Gradient Echo dMRI Sequence Parameters** 3 mm^3^ isotropic resolution, TE = (78, 114, 150, 186) ms, TR = 7.5 ms, coronal plane to maternal habitus. *b* = (5, 10, 25, 50, 100, 200, 400, 600, 1200, 1600) s mm^−2^; 3 directions *b* = 18 s mm^−2^; 8 directions *b* = 36 s mm^−2;^ 7 directions *b* = 800 s mm^−2^; 15 directions

Abbreviation: dMRI, diffusion MRI.

### 2.5 Image reconstruction

The data was processed using in‐house tools, including bias field and motion correction as previously described.^21^ The data acquired at the first TE was motion‐corrected, with the temporal closeness between this and subsequent TEs allowing the transformations required to be applied to all other TEs.

### 2.6 Image analysis

The placental parenchyma was manually segmented on the diffusion images by a clinician experienced in the analysis and segmentation of placental MRI. The clinician performing the segmentation was blinded to the maternal demographics. All subsequent analysis was performed on this masked and motion‐corrected placental diffusion data using in‐house python scripts and our extensions to the diffusion microstructure imaging in python library for diffusion models^23^, ^24^ which enable diffusion–relaxation model fitting.

### 2.7 $T_{2}^{*}\;\mathrm{ADC}$ and $T_{2}^{*}\;\text{IVIM}$ ‐model fitting

Two models were chosen for this study, with both making use of the available combined diffusion multi‐echo data. These include both the simpler biexponential $T_{2}^{*}\;\mathrm{ADC}$ model $T_{2}^{*}\;\mathrm{ADC}$ model: Equation (1); and a bicompartmental $T_{2}^{*}\;\text{IVIM}$ model including both “fast” and “slow” diffusion in two compartments, representing perfusing and diffusing blood within the placenta, $T_{2}^{*}\;\text{IVIM}$ model: Equation (2). The rationale to include the latter more complicated model is the unique perfusion environment in the human placenta, which lends itself ideally for IVIM‐type models.

Equation (1) — $T_{2}^{*}\;\mathrm{ADC}$ model:

(1) $$S\left(T_{E},b\right)=S_{0}e^{-\left(T_{E}-T_{E\min}\right)/T_{2}^{*}e^{-b.\mathrm{ADC}}}.$$

where $T_{E\min}$ is the shortest echo time acquired, *b* is the *b*‐value and S_0_ is the signal at the shortest echo time with zero diffusion weighting.

Equation (2) — $T_{2}^{*}\;\text{IVIM}$ model:

(2) $$S\left(T_{E},b\right)=S_{0}\left[{\mathrm{fe}}^{-b.D*}e^{\left(T_{E}-T_{E\min}\right)/T_{2}^{*P}+\left(1-f\right)e^{-b.\mathrm{ADC}}e^{\left(T_{E}-T_{E\min}\right)/T_{2}^{*D}}}\right].$$

where *S*_0_ is the signal at the lowest TE with zero diffusion weighting, *f* is the perfusion fraction, *b* is the *b*‐value, D* is the pseudo‐diffusion coefficient associated with the perfusion compartment, $T_{E\min}$ is the shortest TE acquired, $T_{2}^{*P}$ is the effective T_2_ associated with the perfusion compartment, ADC is the apparent diffusion coefficient coefficient, and $T_{2}^{*D}$ is the effective T_2_ associated with the diffusion compartment.

We fit the models with a modified version of the diffusion microstructure imaging in python toolbox.^24^ We used the “brute2fine” function, which finds a starting point for the nonlinear optimization by a brute force grid search. For the $T_{2}^{*}\;\text{IVIM}$ model, the ADC values for the fast compartment were restricted to be >0.3 mm^2^ s^−1^, which is the ADC of freely diffusing water.^25^

The $T_{2}^{*}\;\text{IVIM}$ model also produces fractional maps (f) alongside $T_{2}^{*}$ and ADC values for both perfusing and diffusing compartments, representing the fraction of signal from each voxel originating from either the perfusing or diffusing component. These “fractional maps” were multiplied with the corresponding $T_{2}^{*}$ and ADC maps, thus producing $T_{2}^{*}\;\text{IVIM}$ quantities as a weighted sum of the fractions of both compartments in each voxel.

Using these models, and taking into account the perfusing/diffusing compartment fractional maps as described above, values were obtained for all voxels containing placental parenchyma for:


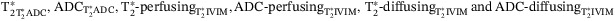
, allowing whole‐placenta maps to be generated for each of these metrics for each MRI scan.

### 2.8 Statistical analysis

Linear regression was used to determine the slope and intercept of mean whole‐placental $T_{2}^{*}$ and ADC values over gestation for all subjects in the cross‐sectional cohort.

Within‐subject rates of change for each measure described above were generated for participants who underwent longitudinal imaging, to determine individual rates of change. The mean and SD of these individual rates of change for $T_{2}^{*}$ and ADC values were then calculated, with covariance being used to describe the overall consistency.

For all analyses, *P* values < 0.05 and absolute covariance values < 1 were considered significant.

## 3 RESULTS

### 3.1 Participant demographics

Data from a total of 17 participants who successfully underwent repeat placental MRI scans during the same pregnancy (longitudinal cohort, 34 scans in total) and 62 participants who successfully underwent a single placental MR scan (cross‐sectional cohort, 62 scans in total) met the study inclusion and image quality criteria described above and were therefore included.

The demographics of the participants in this study are described in Table 2. Both cohorts were comparable in GA, maternal age, and maternal body mass index at the time of the scan for the cross‐sectional cohort, or the average of the two scans for the longitudinal cohort. The average time interval between scans in the longitudinal cohort was 6.87 weeks.

**TABLE 2:** Study demographics

	Longitudinal cohort (17 participants, 34 scans)	Cross‐sectional cohort, (62 participants and scans)
Gestational age at scan (weeks)	30.66 (±4.37)	30.72 (±4.74)
(scan 1 → scan 2)	[27.23 (±3.00) → 34.09 (±2.37)]	–
Maternal age at scan (years)	35.75 (±2.55)	35.28 (±3.88)
(scan 1 → scan 2)	[35.69 (±2.54) → 35.82 (±2.56)]	–
Maternal BMI at scan (kg/m^2^)	25.67 (±3.23)	21.53 (±2.57)
(scan 1 → scan 2)	[25.20 (±3.01) → 26.13 (±3.36)]	–

*Note*: Values reported as (Mean ± SD) unless otherwise stated.

Abbreviation: BMI, body mass index.

### 3.2 Qualitative image data

**FIGURE 2:**
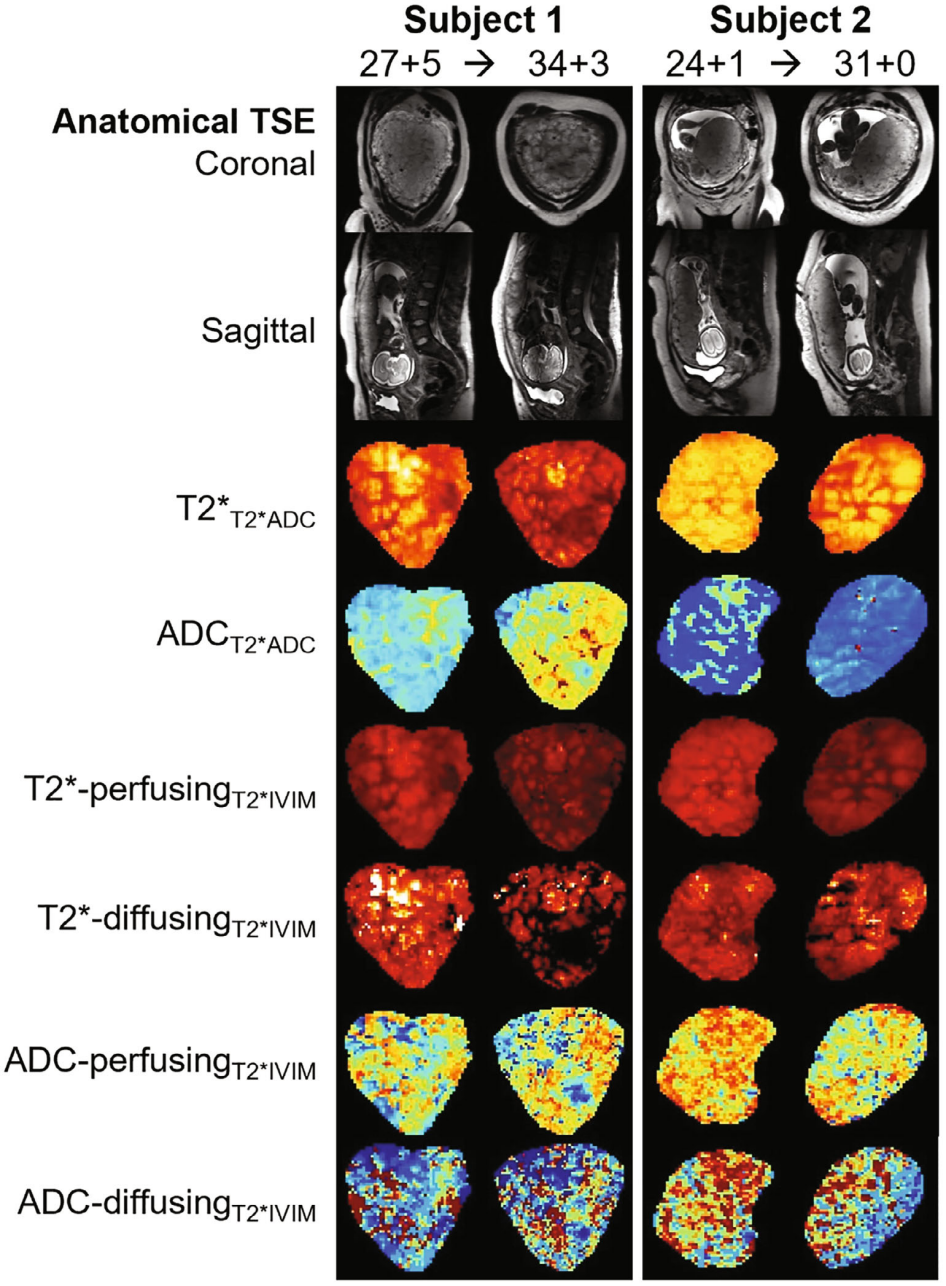
Visual depiction of repeat imaging for two participants from the longitudinal cohort showing anatomical TSE scans in the coronal and sagittal planes, and motion‐corrected diffusion‐weighted imaging, including placental $T_{2}^{*}$ and ADC maps and both $T_{2}^{*}$ and ADC diffusing and perfusing blood maps (coronal slices). For participant 1, scan one was performed at 27^+5^ weeks and scan 2 at 34^+3^ weeks. For participant 2, scan 1 was performed at 24^+1^ weeks and scan 2 at 31^+0^ weeks. TSE, turbo spin echo.

Images from scans performed on two example subjects from the longitudinal cohort are shown in Figure 2, demonstrating the different types of data acquired. These “placental maps” allow an initial qualitative assessment of how placental structure changes over gestation and provide insight into localized changes that occur throughout the placental parenchyma. For example, hypointense rims between the hyperintense lobular structures are visible, along with a general decline in $T_{2}^{*}$ signal as GA increases. Global changes in $T_{2}^{*}$ and ADC values from both diffusing and perfusing compartments are less apparent on these visual maps, although pronounced regional variation is evident, reflecting the underlying heterogeneity of the placental parenchyma.

### 3.3 Quantitative image analysis

The regression results for both the longitudinal cohort specific rates of change (with covariance), and the cross‐sectional cohort linear regression slope (with *R* ^2^ and *P*‐values) are given in Table 3.

**TABLE 3:** Quantitative results from the $T_{2}^{*}$ ADC and $T_{2}^{*}\;\text{IVIM}$ models, reporting both the longitudinal cohort specific rates of change with covariance, and the cross‐sectional cohort linear regression slope *R* ^2^ and *P*‐values

Metrics	Rate of change between visits (longitudinal cohort) Slope mean ± ST (absolute covariance)	Linear regression (cross‐sectional cohort) Slope [*R* ^2^, p]
Mean ${T_{2}^{*}}_{T_{2}^{*}\;\mathrm{ADC}}$	**−3.05 ± 1.44 ms week** ^**−1**^ **(0.47)**	**−2.54 ms week** ^**−1**^ **[0.64, <0.0001]**
Mean ${\mathrm{ADC}}_{T_{2}^{*}\;\mathrm{ADC}}$	−0.05 ± 0.05 mm^2^ s^−1^ week (1.04)	**−0.03 mm** ^**2**^ **s** ^**−1**^ **week** ^**−1**^ **[0.35, 0.0044]**
Mean $T_{2}^{*}$‐${\text{Perfusing}}_{T_{2}^{*}\;\text{IVIM}}$	**−2.71 ± 1.74 ms week** ^**−1**^ **(0.65)**	**−2.35 ms week** ^**−1**^ **[0.57, <0.0001]**
Mean ADC‐${\text{Perfusing}}_{T_{2}^{*}\;\text{IVIM}}$	−0.06 ± 0.08 mm^2^ s^−1^ week^−1^ (1.36)	−0.03 mm^2^ s^−1^ week^−1^ [0.25, 0.052]
Mean $T_{2}^{*}$‐${\text{diffusing}}_{T_{2}^{*}\;\text{IVIM}}$	**−2.71 ± 2.34 ms week** ^**−1**^ **(0.86)**	**−1.91 ms week** ^**−1**^ **[0.38, 0.0084]**
Mean ADC‐${\text{diffusing}}_{T_{2}^{*}\;\text{IVIM}}$	−0.00 ± 0.00 mm^2^ s^−1^ week^−1^ (1.12)	**−0.00 mm** ^**2**^ **s** ^**−1**^ **week** ^**−1**^ **[0.41, 0.0021]**
Perfusion fraction $f_{T_{2}^{*}\;\text{IVIM}}$	0.75 ± 1.93% week^−1^ (2.57)	**0.5% week** ^**−1**^ **[0.35, 0.0017]**

*Note*: Results in **bold** are significant.

### 3.4 $T_{2}^{*}\;\mathrm{ADC}$ model results

**FIGURE 3:**
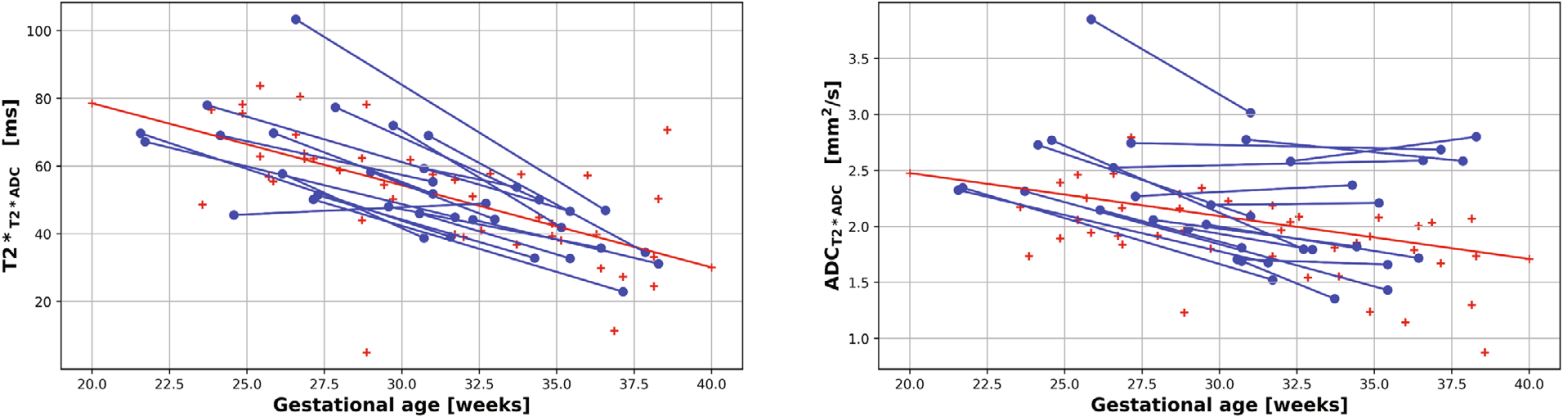
Gestational age at the time of the scan (*x*‐axis) and whole placental $T_{2}^{*}$ values (left) and ADC values (right) from the $T_{2}^{*}$ ADC model (*y*‐axis). The cross‐sectional data points are shown with red crosses and the longitudinal data points with blue dots. The blue lines link measurements for participants who were scanned twice during the same pregnancy (longitudinal cohort). The red line represents the line of best fit, obtained by linear regression, for the cross‐sectional cohort

The linear regression results of the simple $T_{2}^{*}\;\mathrm{ADC}$ model Equation (1) from the cross‐sectional cohort reveal a significant decline in whole‐placental $T_{2}^{*}$ (*R* ^2^ = 0.64, *P* < 0.01) and ADC (*R* ^2^ = 0.35, *P* < 0.01) values over gestation. These declines are consistent within subjects for $T_{2}^{*}$ (−3.057 ± 1.44 ms week^−1^, covariance = −0.47), but not for ADC (−0.05 ± 0.05 mm^2^ s^−1^ week, covariance = −1.04) (Figure 3).

### 3.5 $T_{2}^{*}\;\text{IVIM}$ model results

**FIGURE 4:**
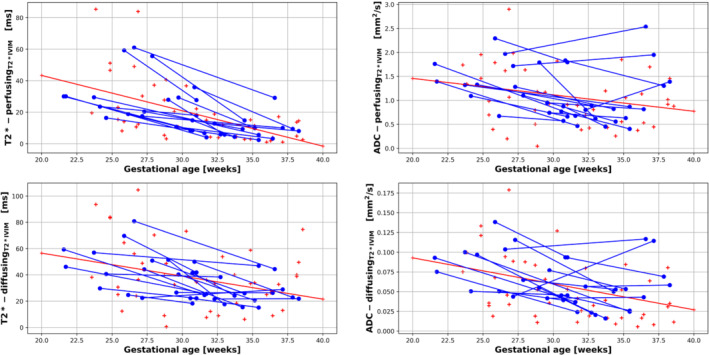
Results from the $T_{2}^{*}\;\text{IVIM}$ model displaying mean placental $T_{2}^{*}$ and ADC values for the perfusing compartment (top row) and diffusing compartment (bottom row). The blue lines link measurements for participants who were scanned twice during the same pregnancy (longitudinal cohort), the red crosses correspond to the cross‐sectional cohort. The red line represents the line of best fit, obtained by linear regression, for the cross‐sectional cohort. IVIM, intravoxel incoherent motion.

**FIGURE 5:**
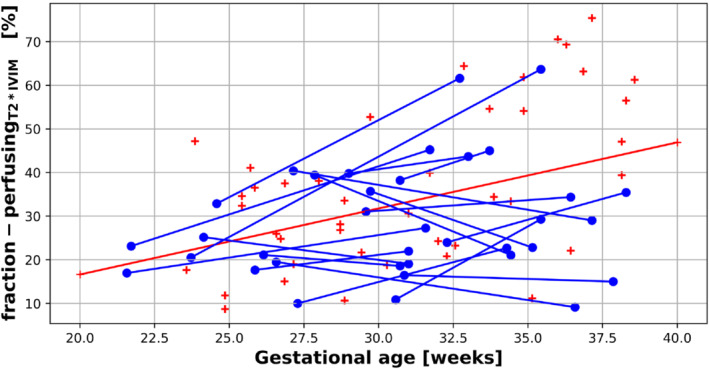
Placental perfusion compartment fraction derived from the $T_{2}^{*}\;\text{IVIM}$ model. The blue lines link measurements for participants who were scanned twice during the same pregnancy (longitudinal cohort), the red crosses correspond to the cross‐sectional cohort. The red line represents the line of best fit, obtained by linear regression, for the cross‐sectional cohort.

The assessment of the $T_{2}^{*}\;\text{IVIM}$ model parameters are shown in Figure 4, with $T_{2}^{*}$ and ADC values from the perfusing compartment in the top two plots, and the diffusing compartment on the bottom two. The fraction of the dMRI signal originating from the perfusing compartment is shown in Figure 5. Quantitative values for all these measurements are shown in Table 3.

In the cross‐sectional cohort, a significant decline in $T_{2}^{*}$ values was demonstrated using linear regression from both the perfusing ($T_{2}^{*}$‐${\text{Perfusing}}_{T_{2}^{*}\;\text{IVIM}}$, *R* ^2^ = 0.57, *P* < 0.001) and diffusing ($T_{2}^{*}$‐${\text{diffusing}}_{T_{2}^{*}\;\text{IVIM}}$ *R* ^2^ = 0.38, *P* = 0.0044) compartments across gestation. This decline in $T_{2}^{*}$ values was consistent within subjects for both the $T_{2}^{*}$‐${\text{Perfusing}}_{T_{2}^{*}\;\text{IVIM}}$ (−2.71 ± 1.74 ms week^−1^, covariance = −0.65) and $T_{2}^{*}$‐${\text{diffusing}}_{T_{2}^{*}\;\text{IVIM}}$ (−2.71 ± 2.34 ms week^−1^, covariance = −0.863) compartments for the longitudinal cohort.

In the cross‐sectional cohort, a significant decline in ADC values was seen using linear regression from the diffusing compartment (ADC‐${\text{diffusing}}_{T_{2}^{*}\;\text{IVIM}}$ *R* ^2^ = 0.41, *P* = 0.0021), but not the perfusing compartment (ADC‐${\text{Perfusing}}_{T_{2}^{*}\;\text{IVIM}}$ *R* ^2^ = 0.25, *P* = 0.052) across gestation. The rates of change of ADC values were not consistent within subjects for either the perfusing compartment (ADC‐${\text{Perfusing}}_{T_{2}^{*}\;\text{IVIM}}$ [−0.06 ± 0.08 mm^2^ s^−1^ week^−1^, covariance = −1.36]) or diffusing compartment  (ADC‐${\text{diffusing}}_{T_{2}^{*}\;\text{IVIM}}$ −0.004 ± 0.004 mm^2^ s^−1^ week^−1^, covariance = −1.12) for the longitudinal cohort.

### 3.6 Placental perfusion fraction results

Using the $T_{2}^{*}\;\text{IVIM}$ model, our results suggest that the fraction of the placenta, which is assumed to contain perfusing blood increases from ∼20% at 20 weeks gestation to almost 50% at 40 weeks, at a rate of 0.5% per gestational week in the cross‐sectional cohort (*R* ^2^ = 0.35, *P* = 0.0017) using linear regression. However, this increase in perfusion fraction was not consistent within subjects (0.75 ± 1.93% week^−1^, covariance = 2.57) (Table 3, Figure 5).

## 4 DISCUSSION

This study represents a comprehensive functional and microstructural placental dMRI assessment in healthy participants at two discrete time points during gestation. It focuses on comparing these longitudinal results with data from a cross‐sectional cohort assessed using the same protocol. It thus bridges an important gap in knowledge by providing data for within‐subject rates of change of placental function and microstructure in a cohort of low‐risk subjects.

### 4.1 $T_{2}^{*}\;\mathrm{ADC}$ changes over gestation

Our results indicate that whole placental $T_{2}^{*}$ values from a joint $T_{2}^{*}\;\mathrm{ADC}$ model, observed in normal, low‐risk pregnancies show consistent decline over gestation, with minimal variation in rates of change in individual pregnancies. Whole placental ADC values derived in the same way appear to show less consistency. Results from the $T_{2}^{*}\;\text{IVIM}$ model indicate that $T_{2}^{*}$ values from both the perfusing and diffusing compartments also decrease consistently across gestation, again, with minimal variation in rates of change in individual pregnancies. Placental ADC values from the perfusing and diffusing components of this joint $T_{2}^{*}$ ADC model are, however, less stable when measured within the same pregnancy.

The obtained quantitative measurements of placental function over GA are in agreement with previous cross‐sectional studies for placental $T_{2}^{*}$ ^10^, ^26^ and ADC,^22^, ^27^ both with regard to the trend over gestation and the reported absolute values of, for example, $T_{2}^{*}$ decay per week at 3 T (Table 3).

Furthermore, the current study used $T_{2}^{*}$ values calculated from an integrated $T_{2}^{*}$‐diffusion acquisition and matched combined $T_{2}^{*}\;\mathrm{ADC}$ model. Whereas studies often rely on multi‐echo gradient echo sequences to generate $T_{2}^{*}$ measurements that are quick (< 1 min) and well established,^10^, ^11^, ^16^, ^28^, ^29^ the combined technique employed here allows simultaneous acquisition of placental $T_{2}^{*}$ and diffusion measures, enabling insight into the complex underlying structure and function of the placenta.^1^, ^14^, ^30^, ^31^

The observed consistency within subjects of whole placental $T_{2}^{*}$ values, from both the $T_{2}^{*}\;\mathrm{ADC}$ and $T_{2}^{*}\;\text{IVIM}$ models, invites speculation as to whether two placentas with a consistent $T_{2}^{*}$ value in the lower range of normal or a consistent $T_{2}^{*}$ value in the higher range of normal vary in terms of anatomical, structural, or vascular properties. This could potentially reflect that the efficiency of oxygen and nutrient transfer is different between individual placentas or imply that combinations of different properties within a placenta may still produce adequate transfer for fetal growth and well‐being, opening up new research avenues to quantify placental capacity in even more detail. It also suggests that even if imaging‐derived metrics of placental function such as $T_{2}^{*}$ values appear to be within normal limits for a specified gestational age, monitoring the rate of change of these metrics within the same pregnancy could provide insight into when placental function might be suboptimal. The longitudinal aspect of this study thus provides potentially a new area for clinical surveillance, namely the assessment of changes in placental function over time.

### 4.2 $T_{2}^{*}\;\text{IVIM}$ results

We successfully deployed a $T_{2}^{*}\;\text{IVIM}$ model, combining the effects of relaxometry with the bicompartmental IVIM model. The $T_{2}^{*}\;\text{IVIM}$ model estimates ADC and $T_{2}^{*}$ values for two separate compartments: fast diffusion (interpreted here as “perfusing”) and slow diffusion (or just “diffusing”). Each compartment is sensitive to different microstructural and circulatory structures, although the extent to which the two compartments have different $T_{2}^{*}$ values remains an open question. Although not reaching statistical significance, our $T_{2}^{*}$ values of the diffusion‐associated compartment are slightly higher than the $T_{2}^{*}$ of the perfusion‐associated compartment, whereas their rates of change over gestation are similar (Figure 4; Table 3). We speculate that the “diffusing” compartment mainly reflects tissues such as the villous tree, structures within placental cotyledons, and water trapped or pooled in small spaces such as the intervillous space, whereas the “perfusing” compartment originates from both the highly oxygenated maternal blood that is streaming relatively quickly from the uterine spiral arteries into the intervillous space, and from the fetal blood perfusing within the fetal vasculature of the placenta,^32^, ^33^ as has been demonstrated in previous work.^1^, ^14^

Similar to the whole‐placental ADC values from the $T_{2}^{*}\;\mathrm{ADC}$ model, our results suggest that rates of change from both the ADC‐${\text{Perfusing}}_{T_{2}^{*}\;\text{IVIM}}$ ADC‐${\text{diffusing}}_{T_{2}^{*}\;\text{IVIM}}$ components of the IVIM model are not consistent within subjects across gestation. This is likely to be partly due to the increased complexity, and therefore error margin, that arises from trying to fit a more complicated bicompartmental model to the dMRI signal, as well as errors arising from partial voluming effects and reduced SNR ratio as a consequence. Recent promising work using the IVIM model to investigate placental vascular malperfusion also found a large variation in intraplacental perfusion fractions in healthy controls,^27^ likely related to the underlying heterogeneity of placental tissue and relatively large voxel size in comparison to the underlying tissue microstructure. Given the voxel size (3 mm^3^ isotropic) used in this study, multiple different tissue types, contributing different amounts of diffusion signal, will often be present in each voxel, which may make measurements more susceptible to external factors and therefore less reliable within subjects. The placental maps shown in Figure 2, highlight the fact that the ADC maps have sharper boundaries between voxels and appear more heterogeneous than the $T_{2}^{*}$ maps.

Given the longer acquisition times required for the diffusion component of the combined $T_{2}^{*}$‐diffusion sequence, the ADC values we have derived here are likely to be more susceptible to motion than $T_{2}^{*}$ values, which may also help explain why ADC values from both the $T_{2}^{*}$ ADC and $T_{2}^{*}\;\text{IVIM}$ models appear to be less consistent when measured within the same pregnancy. The effects of maternal habitus and placental location are also likely to have a more significant effect when more complex models are being used, although these were accounted for in this study.

Considering how placental microstructure develops over gestation may also help explain why greater variation is seen, even within subjects, in the ADC‐perfusing $T_{2}^{*}\;\text{IVIM}$ and ADC‐diffusing $T_{2}^{*}\;\text{IVIM}$ compartment measurements. The ADC signal is highly dependent on whether the movement of water molecules is constrained by certain underlying tissue properties. Whereas remodeling of the uterine spiral arteries, which may affect ADC signal, primarily occurs before the gestational age ranges studied here, the placental villi undergo various changes that could be linked to the variation in ADC values we observe. As the placenta increases in size, the fetal blood vessels entering the villous trees evolve in size and structure and become more branched and dispersed, increasing the surface area available for gas and nutrient exchange.^34^ Their effect on ADC is, however, merely speculative at this point in time. All these changes in underlying placental vascularisation and tissue microstructural properties from within the placental lobules will influence the obtained ADC signal, particularly taking the multiple different spatial directions into account with the chosen *B*‐values and b‐vectors.

Results from the cross‐sectional cohort suggest that the fraction of the placenta composed of “perfusing blood” increases from ∼20% at 20 weeks gestation to ∼50% at 40 weeks gestation, assuming a linear model. Although this increase in perfusion fraction is in line with some recent work,^13^, ^35^ other studies have not shown any significant correlation in placental perfusion fraction with increasing GA, whereas others have identified a negative correlation.^36^ Evidence from ultrasound studies show an increase in placental perfusion with advancing gestational age.^37^ All previous models referenced here were IVIM models and not, as we have used, combined $T_{2}^{*}\;\text{IVIM}$ models. Differences in relaxation times between the two compartments affect the IVIM model parameter estimates similarly to using a different dMRI protocol, as do fitting procedures and region‐of‐interest choices, which may be subtly different between studies. Given there is no significant consistency in the placental perfusion fraction seen *within* subjects in this study (Table 3, Figure 5), and results from other studies have failed to identify a consistent trend in this measurement, this suggests the factors that contribute to the dMRI signal from perfusing and diffusing compartments of the placenta need further investigation, particularly when explored with more advanced models.

### 4.3 Clinical relevance

There is compelling evidence to suggest that placental $T_{2}^{*}$ and ADC values are altered in cases of placental and/or fetal pathology. He et al. have shown that placental $T_{2}^{*}$ values obtained using the IVIM model are significantly reduced in fetuses with IUGR^38^ or those that are small for gestational age.^39^ Similarly, placental ADC values have been shown to be reduced in pregnancies affected by placental dysfunction,^12^ and in fetuses with IUGR.^40^, ^41^ Work using combined placental $T_{2}^{*}$ ADC measurements has also identified differences, when compared to controls, in pregnancies affected by preeclampsia,^1^ chronic hypertension.^42^ Establishing reference ranges for absolute $T_{2}^{*}$ and ADC values of the placenta in normal pregnancy is important when considering how they are affected by placental pathology.

Similarly, whereas work investigating quantitative longitudinal placental $T_{2}^{*}$ values in a large cohort of normal pregnancies has recently been published,^26^ ours is the first study that reports similar longitudinal measurements for both placental $T_{2}^{*}$ and ADC rates of change within individual pregnancies. This provides a useful benchmark against which to compare how joint placental $T_{2}^{*}$ ADC trajectories may change over gestation in pregnancies affected by placental pathology.

### 4.4 Limitations

This study has a number of limitations. Firstly, the repeat scans were not conducted at predefined time windows or with a fixed time between scans. This was partly due to operational constraints with regard to scanner time and participant availability. However, acquiring scans at different intervals enables coverage of a wide GA window and also represents the similarly variable time points during which imaging is performed for assessment in clinical practice. The close similarity between all individual within‐subject rates of change of placental $T_{2}^{*}$ measures demonstrated here suggest that this variation in “time between scans” does not introduce significant bias in this particular metric.

We used data from a cross‐sectional cohort to generate slopes for which to compare within‐subject rates of change of placental $T_{2}^{*}$ and ADC over time. For this, we assumed a linear relationship between both $T_{2}^{*}$ and ADC and changes over gestation, in line with existing literature.^10^, ^11^, ^13^ However, more recent work by Schabel et al. investigating longitudinal $T_{2}^{*}$ placental mapping^26^ suggests that the evolution of placental $T_{2}^{*}$ across gestation is perhaps better described by a sigmoid model. Our study used data acquired from a narrower range of GAs, which may make the application of a linear model for $T_{2}^{*}$ trends appropriate here.

Maternal position in the scanner can impact global and regional changes in placental perfusion^28^, ^43^ and arterial oxygen saturation.^44^, ^45^ As such, this is an important variable to control for. All participants in this study were scanned in the supine position. However, this may restrict the use of the $T_{2}^{*}$ values and ADC measurements reported here as reference values to studies that also perform scans with the mother in a supine position. This could be investigated in future studies.

We used strict maternal inclusion and exclusion criteria, as well as confirming that neonatal outcomes were also normal at the time of writing, to ensure we only included data from low‐risk, healthy control pregnancies. However, placental histology was not available for all of the placentas that were imaged as part of this study, and it is therefore difficult to be certain that they could all be considered macroscopically and microscopically normal once they had been delivered.

Whereas we have explored the use of two different models, we have not attempted to show which one actually explains the data better. However, assessing which model best explains the data does not inform on the clinical utility of the models, which should be assessed independently. Model fits may also be improved by including Rician noise instead of Gaussian.

Finally, this study reports mean quantitative values from the entire placenta. This technique is quick, reliable, and captures useful information that can be helpful for monitoring broad‐scale changes in placental function as pregnancy progresses, or as a tool for identifying potential placental pathology. However, as we have described, the structure of the placenta is not homogenous, and this technique suffers from the fact that it is a largely reductive technique, resulting in the loss of large amounts of potentially useful information. Using descriptives measures of the histograms of all $T_{2}^{*}$ and ADC values for every placental voxel allows the capture of subtle changes in the distribution of these imaging metrics, which can then be interpreted as being representative of both smaller‐scale and regional differences in the biological properties of the underlying placental tissues. Recent related work takes advantage of these approaches, including whole placenta histogram analysis^4^, ^46^ or looking at more focused regions of interest.^19^ Histograms depicting whole‐placenta $T_{2}^{*}$ and ADC voxel values could be useful for visualizing the change in $T_{2}^{*}$ and ADC signal, with the change in shape of these histograms reflecting the change in placental tissue heterogeneity that occurs as the placenta develops.

### 4.5 Future work

The chosen measures and models were limited to $T_{2}^{*}$, $T_{2}^{*}$ ADC, and $T_{2}^{*}\;\text{IVIM}$, and future studies could explore alternative model‐fitting approaches such as Bayesian^47^, ^48^ or machine learning,^49^ or involve a comparison to other placental diffusion–relaxation MRI approaches,^30^ as well as assessing which model best explains the data, for example, by calculating the Bayesian information criterion.

The focus on low‐risk pregnancies was chosen to establish control ranges and to analyze the progression of essential markers in low‐risk healthy placentas. Future work should focus on collecting serial data from high‐risk participants, which can be processed and analyzed using similar techniques. ADC maps are already used to help describe placental heterogeneity and to aid in the characterization of conditions such as placental abruption and gestational trophoblastic disease.^50^ As highlighted earlier, placental ADC and $T_{2}^{*}$ values have been shown to be lower in fetuses with placental insufficiency and IUGR,^1^, ^3^, ^12^, ^38^, ^40^, ^42^ although there is minimal work investigating how these differences evolve over gestation when compared to normal pregnancies.

We have demonstrated that within‐subject rates of change of whole placental $T_{2}^{*}$ and ADC values, derived from a $T_{2}^{*}$ ADC model, are highly consistent, and therefore valid as a measure of placental function and microstructure, with minimal intrasubject variation observed in healthy pregnancies. This implies there might be interesting research avenues into why within‐subject values are as consistent as they are, and what this could tell us about factors influencing placental development at an individual level, as well as potential predictive values of these measurements.

We identified an increase in the fraction of the placenta composed of “perfusing blood” with increasing gestation, although there is a large degree of within‐subject variation in this measurement, consistent with conflicting results from previous studies that have attempted to measure this. More work is needed to understand this.

Whereas the “placental maps” outlined in Figure 2 allow an initial qualitative assessment of how placental structure changes over gestation, future work could involve a more quantitative exploration of variability across the placental parenchyma as a way to assess this systematically, using, for example, texture measures and features to describe heterogeneity in these maps. Furthermore, although two models were investigated here, more complex models are possible and in the future could be accompanied by an analysis of the information content contained in these more complex models. An investigation of how noise in general propagates throughout the data may also prove valuable.

## 5 CONCLUSION

This study provides important data on the evolution of quantitative multi‐modal placental measures over gestation, including, crucially, within‐subject results. A multi‐compartmental $T_{2}^{*}$‐IVIM model was employed, suited to the complex physiology of the human placenta and matched to the used multi‐parametric acquisition technique.

The observed decline in both whole‐placental $T_{2}^{*}$ and ADC values that we observed from the cross‐sectional cohort are in agreement with those seen in other studies.

The greater within‐subject variation observed from the ADC‐${\text{Perfusing}}_{T_{2}^{*}\;\text{IVIM}}$ and ADC‐${\text{diffusing}}_{T_{2}^{*}\;\text{IVIM}}$ compartments is likely to be related to the increased complexity of this model when compared to a simple $T_{2}^{*}$ ADC model, increased susceptibility of ADC measurements to motion, and differences in how certain anatomical regions of the placenta evolve throughout gestation, particularly at a microstructural level.

Finally, the placental rates of change of 3 T dMRI values from normal, low‐risk pregnancies described here may provide a useful benchmark with which to compare other cohorts of interest such as preeclampsia and fetal growth restriction, and in cases of fetal abnormalities such as congenital heart disease.

## FUNDING INFORMATION

This work was supported by grants from the Medical Research Council (MRC) UK, grant (MR/V002465/1); core funding from the Welcome/Engineering and Physical Sciences Research Council (EPSRC) Centre for Medical Engineering, grant (WT203148/Z/16/Z); the National Institutes of Health (NIH), the National Institute of Child Health and Development (NICHD) Human Placenta Project, grant [1U01HD087202‐01]; EPSRC grant EP/V034537/1. j.h. was also supported by a Welcome Trust Sir Henry Welcome Fellowship [201374/Z/16/Z] and a UK Research and Innovation (UKRI) Future Leaders Fellowships (FLF), grant [MR/T018119/1]. The views expressed are those of the authors and not necessarily those of the NHS, the National Institute for Health Research, or the Department of Health.

## Supporting information


**FIGURE S1.** Histograms of voxel values for in‐vivo repeat measurements of whole‐placental ADC (left) and $T_{2}^{*}$ (right). Data from the initial $T_{2}^{*}$‐Diffusion sequence is shown by a solid red line, the repeat $T_{2}^{*}$‐Diffusion sequence is shown by a green dashed line.
**TABLE S1.** In‐vivo repeated measures of placental $T_{2}^{*}$ and ADC values for four participants who underwent repeat diffusion sequences on the same day, during the same scan session.
