# Supplementary material for: Assessing within‐subject rates of change of placental MRI diffusion metrics in normal pregnancy
Source: Magn Reson Med. 2023 May 15;90(3):1137–50. doi: 10.1002/mrm.29665 (PMC10962570; doi:10.1002/mrm.29665)
Supplement: Supplementary file 1 — FIGURE S1. Histograms of voxel values for in‐vivo repeat measurements of whole‐placental ADC (left) and T2* (right). Data from the initial T2*‐Diffusion sequence is shown by a solid red line, the repeat T2*‐Diffusion sequence is shown by a green dashed line. TABLE S1. In‐vivo repeated measures of placental T2* and ADC values for four participants who underwent repeat diffusion sequences on the same day, during the same scan session. [file MRM-90-1137-s001.docx]

# Supplementary Material

## In-vivo repeatability of the T2*-Diffusion sequence

Method:

In order to explore the in-vivo ‘test-retest’ repeatability of the T2*-Diffusion sequence, this particular sequence was repeated at the end of the normal scanning session for four individuals. For one of these participants (GA at scan: 28+5), the T2*-Diffusion sequence was repeated at the end of the normal scanning session, without any other changes, and with the participant remaining in the same position throughout. For two other participants (GA at scan: 29+4 and 25+6), a repeat B0 shim was performed at the end of the normal scanning session immediately prior to a repeat T2*-Diffusion sequence, but again, without changing the position of the participant in the scanner. The fourth participant (GA at scan: 25+3) was removed from the scanner, asked to walk around and then re-positioned in the scanner before a repeat pilot scan, B0 shim, and T2*-Diffusion sequences were performed. This resulted in four ‘paired’ data sets, which were then processed using the same post-processing steps described in the methods section.

This was done to test whether similar values for placental T2* and ADC values would be obtained for each paired data set (i.e. the ‘initial’ T2*-Diffusion sequence and the ‘repeat’ T2*-Diffusion sequence), given that they were acquired in the same participant, in the same supine position, and at the same gestational age and time-of-day for each individual, despite the presence of subtle differences in image acquisition approaches, representing real-world situations that occur where scanning may have to be paused or sequences repeated for e.g. maternal discomfort, and also to account for the fact that steps in the image acquisition and processing pipeline involve non-automated or user-dependent input processes, i.e. B0 shimming or subsequent placental masking, which could introduce non-random variation in results. Only the first T2*-Diffusion sequence acquired on the day of each scan from these four participants were included in the main ‘longitudinal’ cohort analysis, i.e. Data from the repeat T2*-Diffusion sequences for each individual were only used to test in-vivo repeatability of this sequence, as described above, and were not included elsewhere.

Results:

Whole placental T2* and ADC values from the repeat combined T2*-Diffusion sequences acquired for four participants to investigate in-vivo repeatability of this combined T2*-Diffusion sequence and the subsequent image processing pipeline do not show any significant difference in placental T2* or ADC values between the initial and repeat sequences ([Table S1](#sut_invivo_repeatability), [Supplemental Figure 1](#suf_invivo_repeatability)).

| Table S1 In-vivo repeated measures of placental T2* and ADC values for four participants who underwent repeat diffusion sequences on the same day, during the same scan session | | | |
| --- | --- | --- | --- |
| **Metric** | **Initial sequence** | **Repeat sequence** | **T-Test p-value** |
| **T2* (ms) mean ± SD** | 72.7 ± 22.2 | 71.1 ± 25.2 | 0.515 |
| **ADC (mm^2^ s^-1^) mean ± SD** | 3.30 ± 1.31 | 3.34 ± 1.00 | 0.768 |

| 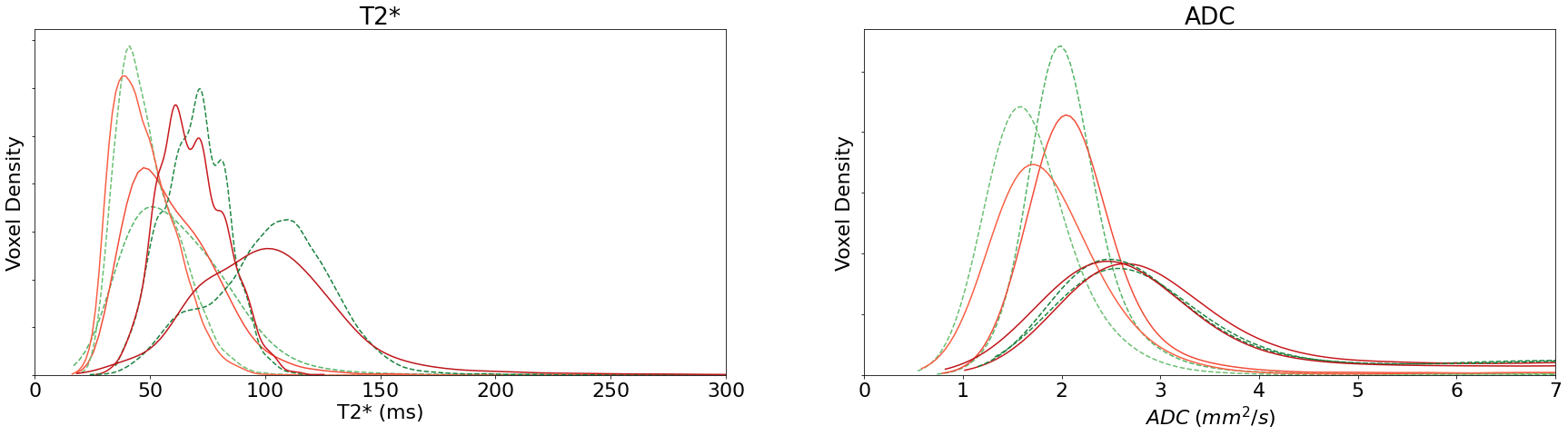 |
| --- |
| Figure S1 Histograms of voxel values for in-vivo repeat measurements of whole-placental ADC (left) and T2* (right). Data from the initial T2*-Diffusion sequence is shown by a solid red line, the repeat T2*-Diffusion sequence is shown by a green dashed line. |
